# MicroRNA-144-3p inhibits autophagy activation and enhances Bacillus Calmette-Guérin infection by targeting ATG4a in RAW264.7 macrophage cells

**DOI:** 10.1371/journal.pone.0179772

**Published:** 2017-06-21

**Authors:** Le Guo, Linlin Zhou, Qian Gao, Aijun Zhang, Jun Wei, Dantong Hong, Yuankui Chu, Xiangguo Duan, Ying Zhang, Guangxian Xu

**Affiliations:** 1 Ningxia Key Laboratory of Clinical and Pathogenic Microbiology, General Hospital of Ningxia Medical University, Yinchuan, China; 2 Department of Medical Laboratory, School of Clinical Medicine, Ningxia Medical University, Yinchuan, China; 3 Clinical laboratory, Affiliated Hospital of north china university of science and technology, Tangshan, China; 4 Department of Molecular Microbiology and Immunology, Bloomberg School of Public Health, Johns Hopkins University, Baltimore, Maryland, United States of America; Univerzitet u Beogradu, SERBIA

## Abstract

MicroRNAs (miRNAs) are small noncoding nucleotides that play major roles in the response of host immune cells. Autophagy plays a key role in activating the antimicrobial host defense against *Mycobacterium tuberculosis* (*M*. *tuberculosis*). Whether miRNAs specifically influence the activation of macrophage autophagy during *M*. *tuberculosis* infection is largely unknown. In the present study, we demonstrate that *Mycobacterium bovis* Bacillus Calmette-Guérin (BCG) infection of macrophages leads to increased expression of miR-144-3p, which targets autophagy-related gene 4a (ATG4a), to inhibit autophagy activation and antimicrobial responses to BCG. Overexpression of miR-144-3p significantly decreased both mRNA and protein levels of ATG4a, inhibited the formation of autophagosomes in RAW264.7 cells and increased intracellular survival of BCG. However, transfection with miR-144-3p inhibitor led to an increase in ATG4a levels, accelerated the autophagic response in macrophages, and decreased BCG survival in macrophages. The experimental results of this study reveal a novel role of miR-144-3p in inhibiting autophagy activation by targeting ATG4a and enhancing BCG infection, and provide potential targets for developing improved treatment.

## Introduction

*Mycobacterium tuberculosis* (*M*. *tuberculosis*), the causative agentof infectious tuberculosis, is a major human pathogenic bacterium that can survive within macrophages, and leads to 1.5 million people death every year in the world [1]. A coordinated cell response mediated by macrophages and T lymphocytes from the innate and adaptive immune system is required for the control of *M*. *tuberculosis* infection [2]. Interestingly, macrophages are not only primary cell types in which *M*. *tuberculosis* resides predominantly, but also the first line of defense against *M*. *tuberculosis* [3]. The recognition of *M*. *tuberculosis* is mediated by various pattern recognition receptors (PRRs). Interaction of mycobacterial ligands with PRRs leads macrophages and dendritic cells (DCs) to secrete selected cytokines which can play a protective role against *M*. *tuberculosis* [4]. However, *M*. *tuberculosis* can evade destruction by antimicrobial defense mechanisms of the innate immune system, causing survival and persistence of *M*. *tuberculosis* within host macrophages [5]. For instance, *M*. *tuberculosis* can prevent maturation of normal phagosome, block phagosome fusion with lysosomes, and produce a favorable environment for bacterial survival and replication [6, 7]. Therefore, a critical balance in the interactions between the macrophage and *M*. *tuberculosis* could be instrumental in determining the outcome of infection. During the co-existence with host, *M*. *tuberculosis* consist of a complex network within macrophages to manipulate the immune response for its survival [8].

Autophagy is an evolutionarily conserved mechanism of cellular self-digestion in which damaged organelles and misfolded proteins are degraded in order to recycle nutrients for cell survival [9, 10]. Increasing evidence has suggested that autophagy is a central element of innate immune responses against different invading pathogens [11]. Besides, autophagy is regulated primarily by numerous distinct autophagy-related (ATG) proteins. Thus far 34 ATGs in all subtypes of autophagy have been identified [12, 13], such as ATG1, ATG4, ATG5, ATG12 and ATG8. Among the ATGs, ATG4 and microtubule-associated protein light chain 3 (LC3) are essential for autophagy induction. ATG4 enzymes can cut off C-terminal last five amino acids of LC3 and expose a critical glycine residue of LC3. In addition, ATG4 proteins also contribute to the deconjugation reaction of LC3-II, as ATG4 proteins delipidates LC3-II and removes it from the autophagic vesicle surface [14, 15]. The deconjugation of LC3 regulated by ATG4a proteins was widely regarded as a very important event influencing the amplitude of the autophagic response [16].

MicroRNAs (miRNAs) are a class of evolutionarily conserved, endogenous, single stranded RNA molecules containing about 22 nucleotides and post-transcriptionally regulate cellular gene expression through partial binding to the mRNA of genes. MicroRNAs regulate gene expression by a variety of mechanisms [17]. In addition, miRNAs are known to be involved in various biological pathways, including differentiation, development, cell growth, immune regulation, apoptosis, and disease progression [18]. Recently, miRNAs have emerged to be involved in immune responses of host cells against invading pathogens [19]. They also acted as important regulators of autophagy, which is medicated by lysosome and aided in degradation of a cell’s own compoments [20–24].

The miR-144-3p belongs to the cluster miR-144/451 or miR-144 family which encodes these miRNAs (miR-144-3p, miR-144* and miR-451). Research has shown that cardiac hypertrophy and cardiac autophagy were regulated by miR-451, which can target TSC1 [25]. The formation of autophagosome was depressed by overexpression of miR-451 *in vitro*. Conversely, autophagosome formation was accelerated by miR-451 knockdown. Another study confirmed that the expression levels of sputum and serum miR-144-3p were significantly upregulated in the tuberculosis patients, but were found to decrease significantly after anti-tuberculosis treatment [26]. In addition, it has been reported that cisplatin-induced VEGF can suppress the expression levels of miR-144 in prostate cancer cells, which subsequently upregulates Beclin-1 to increase autophagic cell survival against cisplatin-induced cell death [27]. Chen S et al have shown that miR-144 increased autophagy in lung cancer cells by targeting TIGAR [28]. However, the regulatory function of miR-144-3p in intracellular bacterial clearance mediated by autophagy remains unclear.

In the present study, we explored the potential role of miR-144-3p in RAW264.7 macrophages in response to Bacillus Calmette-Guérin (BCG) infection. We found that miR-144-3p expression was significantly induced in macrophage RAW264.7 cells after BCG infection. Overexpression of miR-144-3p reduced the abundance of ATG4a, p62 and LC3I/II protein, thus inhibiting maturation of autophagosome in macrophages. Furthermore, we confirmed that ATG4a is a novel functional target of miR-144-3p in inhibiting autophagy activation and enhancing mycobacterial infection. These findings contribute to a better understanding of mechanisms that facilitate bacterial survival and replication in macrophages.

## Materials and methods

### Cells and mycobacterial culture

Murine macrophage RAW264.7 cells (ATCC; TIB-71) were purchased from the Type Culture Collection of the Chinese Academy of Sciences, Shanghai, China. The cells were cultured at 37℃ in a humidified atmosphere of 5% CO_2_ and 95% oxygen. They were maintained in RPMI-1640 or DMEM medium (Gibco) supplemented with 10% Fetal Bovine Serum (FBS) and 1% pen/strep. Bacillus Calmette-Guérin (BCG) Beijing strain developed from BCG Denmark, which shows protective efficacy similar to that of BCG Denmark [29], was provided by Chinese Center for Disease Control and Prevention (CCDC). *M*. *bovis* BCG cultures were grown in Middlebrook 7H9 broth supplemented with 10% albumin dextrose catalase (ADC) for 2 weeks. The precipitates were harvested by centrifugation and and resuspended in the BCG culture medium.

### Quantitative real-time PCR (qRT-PCR)

For qRT-PCR analysis, TRIzol reagent (Sigma-Aldrich, T9424) was used to isolate Total RNA. RNAiso for Small RNA kit (Takara) was utilized to purify miRNAs. The miR-144-3p levels were assessed by qRT-PCR by using TransStart Top Green qPCR SuperMix kit (TransGen Biotech) with thermal cycling conditions of 95℃ for 30 s, followed by 50 cycles of 95℃ for 5 s, 60℃ for 20 s, and 72℃ for 20 s. Small nuclear RNA (RNU6) was used to normalize for miR-144-3p. The primer sequences for miR-144-3p quantification were as follows: miR-144-3p for cDNA reverse transcription (5’-CTCAACTGGTGTCGTGGAGTCGGCAATTCAGTTGAGAGTA CATC-3’), miR-144-3p for qRT-PCR (Forward, 5’-ACACTCCAGCTGGGTAC AGTATAGATGATGTACT-3’; Reverse, 5’-CTCAACTG GTG TCGTGGA-5’).

In order to quantify the BCG burden in RAW264.7 cells following a treatment, a qRT-PCR assay was employed [30]. Briefly, the RAW264.7 cells were transfected with miR-144-3p control, miR-144-3p mimic or inhibitor for 24 h, or were treated with rapamycin or 3-MA for 2 h. After that, the RAW264.7 cells were infected with BCG for 24 h. The cells were then harvested for total DNA extraction using a commercially available genomic DNA kit (Tiangen Biotech, Beijing, China). The abundance of intracellular bacilli was determined by accessing BCG DNA using a qPCR assay; the primer set used for qPCR was based on BCG specific sequence (IS6110, Sequence ID: gi|6006564|emb|X57835.2|); the sequences of primers were F: 5’-GGACGGAAACTTGAACACG-3’ and R: 5’-TCTGACGACCTGATGATTGG-3’. Standard PCR cycle parameters were as follows: 95℃ for 30 s, followed by 40 cycles of 95℃ for 15 s, 60℃ for 30 s and 72℃ for 30 s. The BCG abundance was calculated against β-actin (5’-CAAGTCATCACTATTGGCAACGA-3’; R: 5’-CCAAGAAGGAAGGCTGGAAAA-3’), an internal control of host cells, using a comparative Ct (ΔΔCt) method.

### Transient transfection

To determine the potential function of miR-144-3p, RAW264.7 cells were transiently transfected with 50 nM control or miR-144-3p mimic (GenePharma, Shanghai, China); 50 nM control or miR-144-3p inhibitor (GenePharma, Shanghai, China) by using Lipofectamine 2000 (Invitrogen, USA).

### Target prediction and dual-luciferase reporter assay

The targets of miR-144-3p were identificated by bioinformatic software, miRanda (http://www.microrna.org/microrna/home.do) online, DIANA-MICROT (http://diana.cslab.ece.ntua.gr) and miRDB (http://mirdb.org/miRDB/). For dual-luciferase reporter assay, the ATG4a 3' UTR segments, containing the binding elements of miR-144-3p or its mutant versions were synthesized as sense and antisense linkers. The primers used during the study were ATG4a primers and ATG4a mutant primers, as shown in S1 Table. The mutated nucleotides in the primer sequence were indicated by bold. Besides, the restriction sites of *Mlu I* and *HindIII* were respectively introduced in the forward and reverse primers and indicated by underline. The cDNA generated from RAW264.7 RNA was used as templates for amplification of the 3’UTR fragment by a PCR assay. The wild-type and mutated 3’UTR fragment were then cloned into the downstream of luciferase reporter gene of pMIR-Report vector (Promega, Madison, WI, USA), by which the ATG4a mRNA luciferase reporter constructs, pMIR-Report-WT-ATG4a (harboring wild-type 3’UTR) and pMIR-Report-Mut-ATG4a (harboring mutant 3’UTR) were generated. The specificity of miR-144-3p targeting ATG4a mRNA was ascertained by co-transfection of miR-144-3p mimic or inhibitor (Shanghai GenePharma Co.,Ltd) and pMIR-Report-ATG4a/Mut into RAW264.7 cells, and determined by relative activity of firefly luciferase unit (RLU) at 48 h post-transfection using a dual-luciferase reporter assay kit as recommended by the manufacturer (Promega, Madison, WI, USA). A Renilla luciferase expressing plasmid pRL-TK (Promega, Madison, WI, USA) was included in the transfection to normalize the efficiency of each transfection.

### Immunoblotting analysis

The cellular total protein were obtained by a protein extraction kit. Besides, the Bradford protein assay kit (BESTBIO) was used to determine the protein concentration. 30 μg or 50 μg of total cell extracts were analyzed by Western blotting. The proteins were loaded onto 15% SDS-PAGE gels and transferred onto a polyvinylidene difluoride (PVDF) membrane (Millipore, USA). Membranes were incubated with 1 μg/ml Rabbit anti-ATG4a antibody (Polyclonal, ABGENT), 2 μg/ml mouse anti-SQSTM1/p62 antibody (Monoclonal, Abcam), 2 μg/ml rabbit anti-LC3 antibody (Cell Signaling Technology, Inc.), 2.5 μg/ml rabbit anti-GAPDH antibody (Monoclonal, Cell Signaling Technology, Inc.) or 2 μg/ml mouse anti-β-actin antibody (Monoclonal, Cell Signaling Technology, Inc.). After that, the membranes were incubated with 1 μg/ml HRP-conjugated goat anti-rabbit IgG secondary antibody or 1 μg/ml HRP-conjugated goat anti-mouse IgG secondary antibody (Proteintech Group, Inc.). Immunoreactive band analysis was performed by using the enhanced western bright ECL reagent (Advansta, United States). Densitometry analyses of western blots were performed with ImageJ software (version 1.46). Western blots were developed to be linear in the range used for densitometry. All results were expressed as a relative ratio to the β-actin or GAPHD. At least three or four independent western blot experiments were performed.

### Immunocyto-fluorescent staining

The RAW264.7 cells were transfected with miR-144-3p control, miR-144-3p mimic or miR-144-3p inhibitor for 24 h, or treated with 50 μg/ml rapamycin (MedChemExpress) in 0.1% DMSO. After that, the RAW264.7 cells were infected with BCG for 24 h. The cells were fixed in 4% paraformaldehyde for 20 min, permeabilized with 0.2% Triton X-100 (Sigma) for 10 min, and incubated with 10 μg/ml rabbit anti-LC3 primary antibody (Cell Signaling Technology, Inc.) at 4℃ for overnight. The cells were then incubated with 2 μg/ml Alexa Fluor 488–conjugated goat anti-rabbit IgG (Abcam) at room temperature for 1 h. Nuclei were stained with Prolong Gold anti-fade reagent with 4’-6-diamidino-2-phenylindole (DAPI) (P36931, Invitrogen, Grand Island, NY, USA) for 1 min. The fluorescence images were acquired using an Olympus DSU spinning disk confocal microscope under a 100 × objective oil lens. To quantify autophagy, endogenous LC3 punctate dots were determined using ImageJ software (version 1.46). At least 15 cells were counted per condition in each experimental group and assays were repeated in triplicate for each condition.

### Fluorescent dye Lyso tracker RED labeled acid compartments

RAW264.7 cells infected with BCG were transiently transfected with control, miR-144-3p mimic, miR-144-3p inhibitor, or treated with 20 μM chloroquine (CQ, Sigma) or 50 μg/ml rapamycin (MedChemExpress). Afte that, the RAW264.7 cells were incubated with a specific fluorescent dye Lyso tracker RED (50 nM) for 2 h at 37℃. The cells were then washed to remove excess label. The Lyso tracker RED labeled autophagic vacuoles were detected by confocal microscopy.

### Transmission electron microscopy

The detection of autophagosomes was performed by transmission electron microscopy (TEM) according to the methods as previously reported [30, 31]. The RAW264.7 cells were transfected with miR-144-3p control, miR-144-3p inhibitor or mimics, or were treated with 50 μg/ml rapamycin (MedChemExpress) or rapamycin plus miR-144-3p mimic, and the infected with BCG for 24 h. After fixed in 2% v/v glutaraldehyde in 0.05 M sodium phosphate buffer for 24 h, the samples were subsequently postfixed in 1% w/v OsO4 for 2 h. After dehydration with ethanol, the samples were transferred to propylene oxide and embedded in Epon. The sections (80 nm) were stained with uranyl acetate and lead citrate, and examined using a Phillips CM 100 BioTWIN transmission electron microscope (magnifications: ×1000 for overviews and ×3000 for close-ups). The highresolution images of cellular cross-sections were obtained by using ITEM digital imaging software. For each experimental group, the autophagosomes of 15 cellular cross-sections were counted.

### Colony-Forming Unit assay

Colony-Forming Unit (CFU) assay was performed according to the method described previously with some modifications [23, 32]. RAW264.7 cells were transfected with miR-144-3p control, miR-144-3p mimic or miR-144-3p inhibitor for 24 h, or treated with rapacmycin (50 μg/ml) or 3-MA (10 mM) for 2 h, and then infected with 1×10^7^ BCG. After incubation with BCG for 1 h at 37℃, the extracellular mycobacteria were removed by washing with PBS. The infected cells were cultured for anther 24 h, 48 h, or 72 h. After that, quantitative culturing was performed using 10-fold serial dilutions on Middlebrook 7H10 agar plates with OADC. Plates were cultured for 3 weeks, and colonies were counted.

### Statistical analysis

Statistical analyses were performed using two-tailed Student’s *t*-test. Comparisons between groups were performed using ANOVA. Data was presented as the mean standard deviation SD). Significant differences were assigned to p values <0.05, <0.01 and <0.001, denoted by *, **and ***, respectively.

## Results

### MiR-144-3p is significantly increased in RAW264.7 after BCG infection

**Fig 1:**
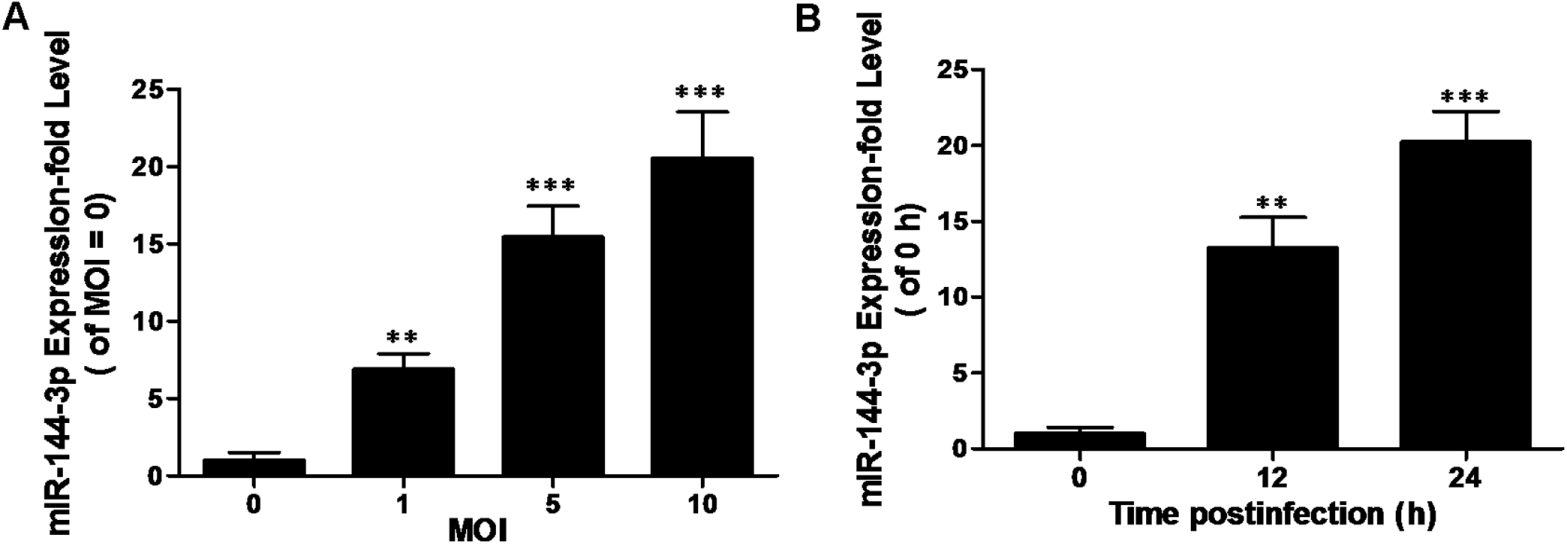
miR-144-3p expression is induced after BCG infection. **(A)** RAW264.7 cells were infected with BCG at different MOI for 24 h, and the expression levels of miR-144-3p were measured by qRT-PCR. The normalized miR-144-3p expression for control (MOI = 0) was set as 1. Data are shown as the mean ± SD of at least three independent experiments. **, p<0.01; ***, p <0.001. **(B)** miR-144-3p expression levels were examined after infected with BCG at an MOI of 10 for the indicated times, and the expression levels of miR-144-3p were measured by qRT-PCR. The normalized miR-144-3p expression for control (the infection time = 0) was set as 1. Data are shown as the mean ± SD of at least three independent experiments. *, p <0.05; **, p<0.01; ***, p <0.001.

MiR-144-3p plays essential regulatory roles in the host immune response [33, 34]. However, little is known about its functional role in modulating autophagy. Recent studies have revealed that the expression levels of sputum and serum miR-144 were significantly upregulated in the tuberculosis patients, but were found to decrease significantly after anti-tuberculosis treatment [26]. These data imply a potential correlation of miR-144-3p with mycobacterial infection. In order to identify miR-144-3p expression level *in vitro* in response to mycobacterial infection, the expression level of miR-144-3p was detected in the RAW264.7 infected with BCG. Using a qRT-PCR assay, we found that the expression level of miR-144-3p was increased in BCG-challenged RAW264.7 cells in a dose- and time-dependent manner (Fig 1A and 1B). Our results indicate that the miR-144-3p could play a regulatory role in macrophages in response to mycobacterial infection. Besides. miRNA expression profile in dendritic cells (DCs) infected with BCG wes also identified by microarray analysis. The result showed that miR-144-3p expression was up-regulated in DCs infected with BCG, as shown in S1 Fig and S1 Text.

### MiR-144-3p suppresses ATG4a by interacting with its 3'UTR

**Fig 2:**
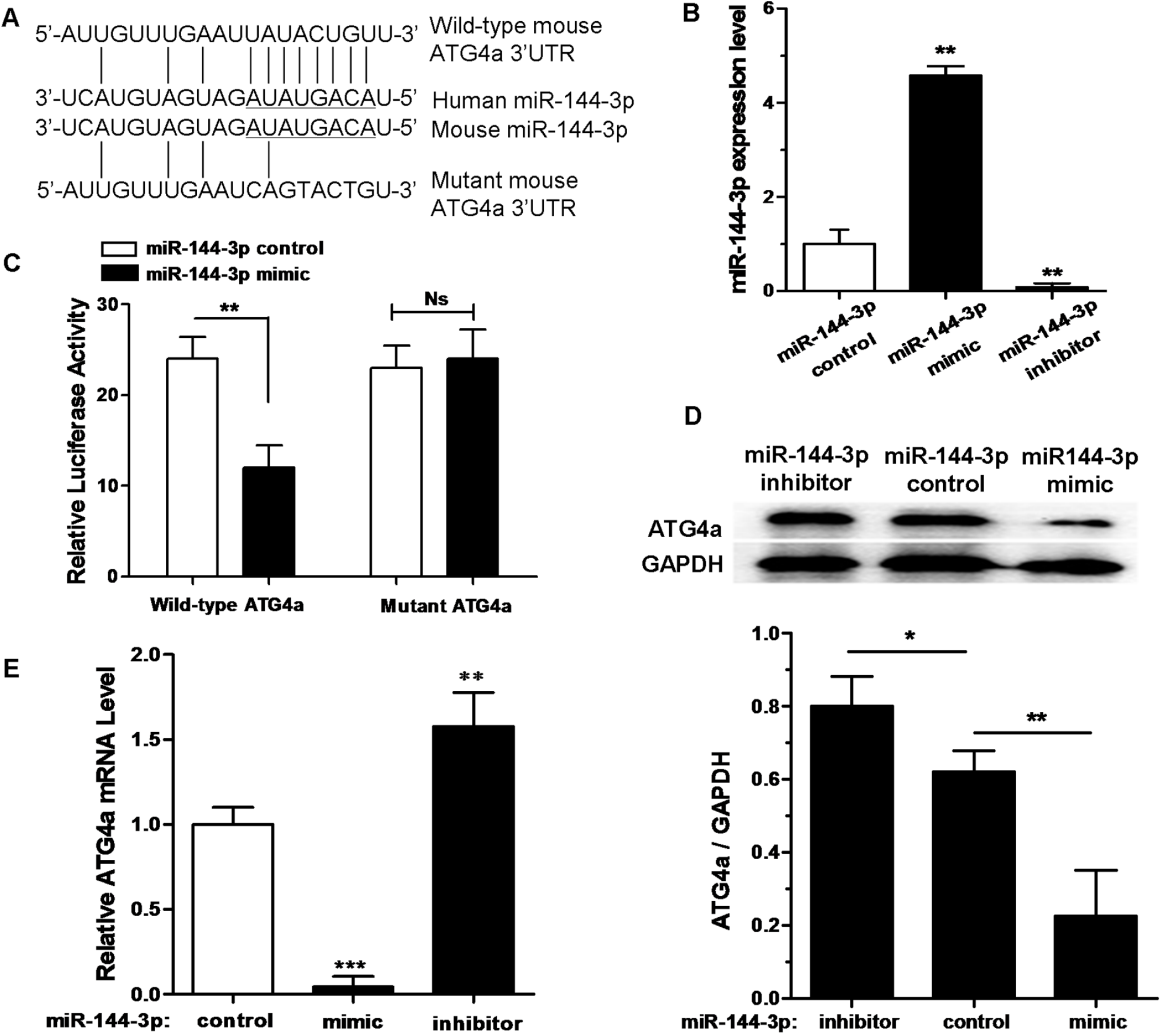
miR-144-3p represses ATG4a expression by targeting its 3' UTR. **(A)** Bioinformatic analysis was used to predict the mouse miR-144-3p seed sequences and the 3'UTR sequences of wild type (WT) and mutant ATG4a. **(B)** The expression level of miR-144-3p in RAW264.7 cells transfected with miR-144-3p control, miR-144-3p mimic or its inhibitor was determined by a qRT-PCR assay. qRT-PCR data were normalized with U6 small nucleolus RNA data. Data represent the mean ± SD from three independent experiments. **p<0.01. **(C)** RAW264.7 cells were cotransfected with pMIR-REPORT Luciferase vector carrying wild-type ATG4a or mutant ATG4a, along with miR-144-3p control or miR-144-3p mimics, and a luciferase assay was performed. Data are shown as mean ± SD of four independent experiments. **p<0.01. **(D)** RAW264.7 cells were transfected with miR-144-3p control, miR-144-3p mimic or miR-144-3p inhibitor. 50 μg of total cell extracts were analyzed by Western blotting with a rabbit anti-ATG4a antibody. Representative Western blot image for ATG4a expression and quantitative analysis of ATG4a protein levels, normalized to GAPDH expression, are shown. Data are shown as mean ± SD of four independent experiments. *, p <0.05; **, p<0.01. **(E)** RAW264.7 cells were transfected with miR-144-3p control, miR-144-3p mimic or miR-144-3p inhibitor. The expression levels of ATG4a mRNA were detected by qRT-PCR. Data represent the mean ± SD from three independent experiments. **p<0.01, ***p<0.001.

Autophagy is a defense mechanism suppressing intracellular bacteria survival in macrophages [35]. Based on bioinformatic analysis by miRanda, DIANA-MICROT and miRDB, the relevant targets of miR-144-3p which regulates autophagy were identified, and then its interaction network was drawn (data not shown). We found that ATG4a, which was known as an important protein which can influence autophagic response, displayed a potential target sequence matching with miR-144-3p in its 3'UTR (Fig 2A). Real-time PCR data displayed that miR-144-3p mimic could increase the miR-144-3p expression level. However, miR-144-3p inhibitor could markedly reduce the expression level of miR-144-3p in RAW264.7 cells (Fig 2B). The dual luciferase data showed that miR-144-3p over-expression could inhibit luciferase activity in the RAW264.7 cells containing the WT-ATG4a 3'UTR reporter. However, miR-144-3p overexpression did not have a significant inhibition effect on luciferase activity in the RAW264.7 cells containing Mut-ATG4a 3'UTR reporter (Fig 2C). The quantitative densitometric data analysis from the proteins on the Western blot showed that transfection with an miR-144-3p mimic resulted in a significant decrease in ATG4a protein expression in RAW264.7 cells. In contrast, ATG4a protein levels were significantly increased in RAW264.7 cells, after endogenous miR-144-3p was blocked by the transfection of an miR-144-3p inhibitor (Fig 2D). Besides, the expression level of ATG4a mRNA was suppressed by miR-144-3p mimics (Fig 2E). However, miR-144-3p inhibitors had an inducing effect on the levels of ATG4a mRNA expression. Taken together, these results indicate that miR-144-3p inhibits ATG4a expression by targeting its 3'UTR binding site.

### MiR-144-3p inhibits autophagy in macrophages

**Fig 3:**
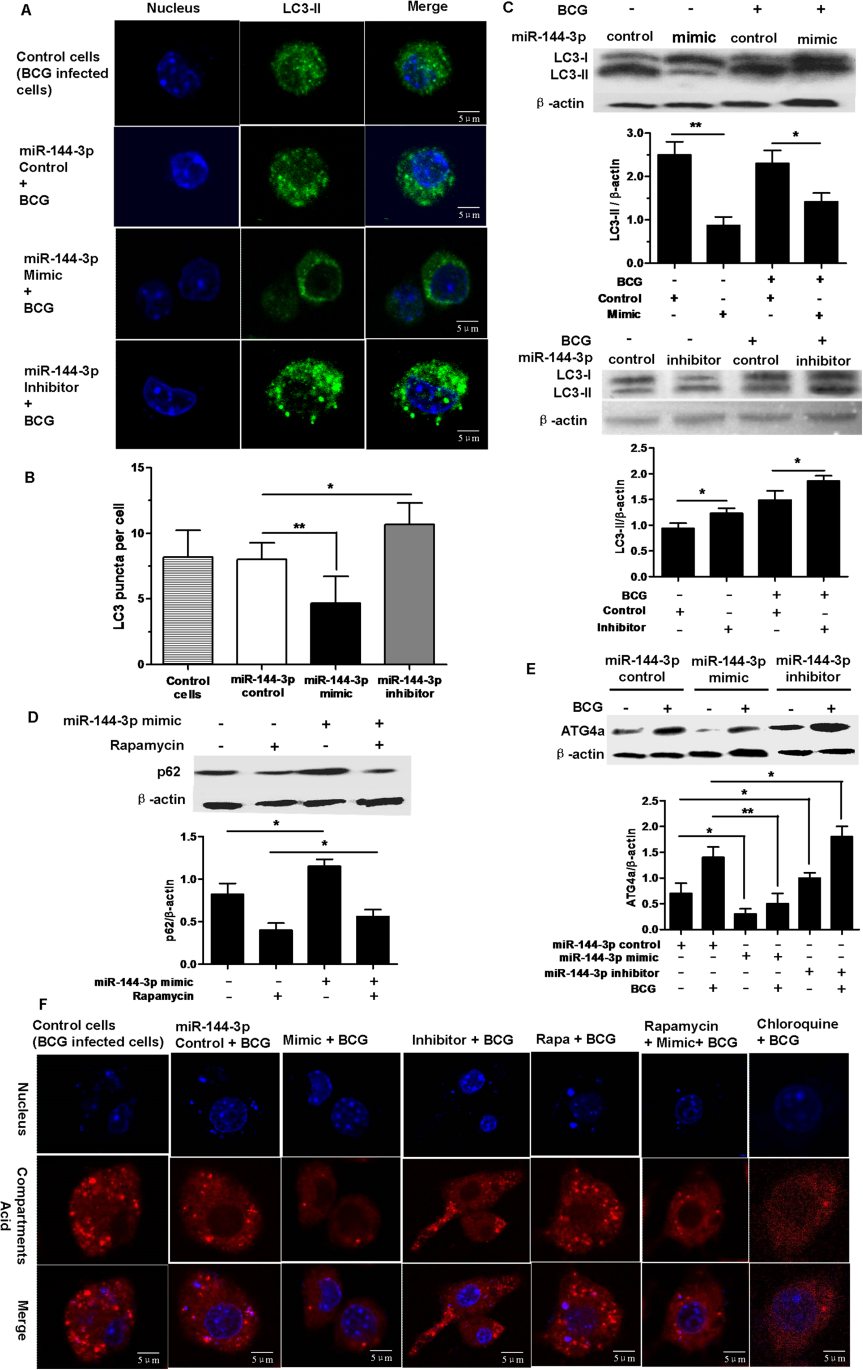
miR-144-3p inhibits autophagy in RAW264.7 cells. **(A and B)** RAW264.7 cells were transfected with miR-144-3p control, miR-144-3p mimic or inhibitor, and then infected with BCG for 24 h. The cells were incubated with a rabbit anti-LC3 Ab, followed by detection using Alexa Fluor 488–conjugated goat anti-rabbit IgG and DAPI. The formation of LC3 puncta was then detected by confocal microscopy. Scale bar = 5 μm. The control cells are BCG-infected cells without transfection with any types of miRNAs. LC3 puncta were determined using ImageJ software (miR-144-3p control, *n* = 15 cells; miR-144-3p mimic, *n* = 15 cells; miR-144-3p inhibitor, *n* = 15 cells). Data represent the means ± SD from three independent experiments. *p < 0.05, **p < 0.01. **(C)** RAW264.7 cells were transfected with an miR-144-3p mimic or inhibitor and then infected with BCG for 24 h. 30 μg of total cell extracts were analyzed by Western blotting with a rabbit anti-LC3 antibody. The conversion of LC3-I to LC3-II was determined by Western blot analysis (upper). The quantitative analysis of LC3-II protein levels, normalized to β-actin expression, are shown (lower). Data are shown as mean ± SD of three independent experiments. *, p <0.05; **, p<0.01. **(D)** RAW264.7 cells transfected with miR-144-3p control or miR-144-3p mimic in full medium with or without 50μg/ml rapamycin or 0.1% DMSO. 50 μg of total cell extracts were analyzed by Western blotting with a mouse anti-SQSTM1/p62 antibody. The p62 levels were detected by Western-blot (upper). Quantitative analysis of the p62 band normalized to β-actin is shown (lower). Data are shown as mean ± SD of four independent experiments. *, p <0.05. **(E)** RAW264.7 cells were transfected with miR-144-3p control, miR-144-3p mimic or inhibitor and then infected with BCG. 50 μg of total cell extracts were analyzed by Western blotting with a rabbit anti-ATG4a antibody. Representative Western blot image for ATG4a expression (upper) and quantitative analysis of ATG4a protein levels, normalized to β-actin expression (lower), are shown. Data are shown as mean ± SD of three independent experiments. *, p <0.05; **, p<0.01. **(F)** miR-144-3p inhibited autolysosome formation. RAW264.7 cells were transiently transfected with miR-144-3p control, miR-144-3p mimic or inhibitor, or were treated with rapamycin, chloroquine, or rapamycin plus miR-144-3p mimic. Then RAW264.7 cells were infected with BCG for 24 h. After that the RAW264.7 cells were incubated with a specific fluorescent dye Lyso tracker RED (50 nM). The control cells are BCG-infected cells without transfection with any types of miRNAs. The positive acid compartments were detected by confocal microscopy. Scale bar = 5 μm.

ATG4a and LC3 play essential roles in the initiation of autophagy [36]. Because ATG4a is a target protein of miR-144-3p, we determine whether miR-144-3p regulated the activation of autophagy in macrophages during BCG infection. To test the hypothesis that miR-144-3p inhibits autophagy in macrophages to arrest the maturation of mycobacterial phagosomes, we evaluated the autophagic activity in RAW264.7 cells using Western blotting and confocal microscopy to test the processing of LC3 (conversion from LC3-I to LC3-II) and the number of LC3-II puncta, respectively. Results showed that miR-144-3p mimics inhibited the formation of LC3 puncta. However, miR-144-3p inhibitors promoted the formation of LC3 puncta (Fig 3A and 3B). Western-blot results showed that the expression level of LC3-II was decreased in uninfected or BCG-infected RAW264.7 cells after transfection with miR-144-3p mimics, but LC3-II expression level was increased in BCG-infected RAW264.7 cells after transfection with miR-144-3p inhibitors (Fig 3C). These results indicated miR-144-3p had an inhibitory effect on autophagy in RAW264.7 cells.

In order to further determine the inhibitory effect of miR-144-3p on autophagy, the expression level of p62 protein, which is a molecular carrier of cargo to be degraded by autophagy [37], was measured by Western blotting. When autophagy is induced, the p62 protein level is decreased, and the p62 protein level is increased when autophagy is inhibited. Thus, the p62 is a relatively ideal marker to explore autophagic flux. We found that miR-144-3p overexpression could increase the expression level of p62 in RAW264.7 cells transfected with or without rapamycin (Rapa) which is an autophagy inducer, indicating that miR-144-3p had an inhibition of autophagy. Furthermore, the inhibitory effects of miR-144-3p on autophagy in RAW264.7 cells were partly reversed by treatment with Rapa, as indicated by Western-blot (Fig 3D), revealing that miR-144-3p had antagonistic action on the induction of autophagy by Rapa. To establish the specificity of miR-144-3p, we further examined whether overexpression of miR-144-3p can decrease ATG4a protein expression in uninfected and BCG–infected RAW264.7 cells. The results showed that transfection with an miR-144-3p mimic resulted in a significant decrease in ATG4a protein expression in uninfected and BCG–infected RAW264.7 cells. In contrast, transfection with an miR-144-3p inhibitor increased ATG4a protein expression in uninfected and BCG–infected RAW264.7 cells (Fig 3E).

To further confirm that miR-144-3p can inhibit autophagy, we labeled and tracked acidic organelles by using LysoTracker Red. Lysotracker Red can label acid compartments including degradative autophagic vacuoles (autolysosomes) and lysosomes. Compared with BCG-infected RAW264.7 cells transfected with miR-144-3p control, there were less red spots in BCG-infected RAW264.7 cells after transfection with miR-144-3p mimics or chloroquine (CQ) which is an autophagy inhibitor, indicating that overexpression of miR-144-3p could inhibit the formation of acid compartments just as CQ could (Fig 3F). Besides, the miR-144-3p inhibitors just like rapamycin which is an autophagy inducer could promote the formation of acid compartments. Furthermore, the inhibitory effects of rapamycin on the formation of acid compartments were partly reversed by miR-144-3p mimic, revealing that miR-144-3p had antagonistic action on the induction of autophagy by rapamycin. In a few words, these results implied that miR-144-3p could suppress autophagic response.

### MiR-144-3p inhibits autophagosome formation

**Fig 4:**
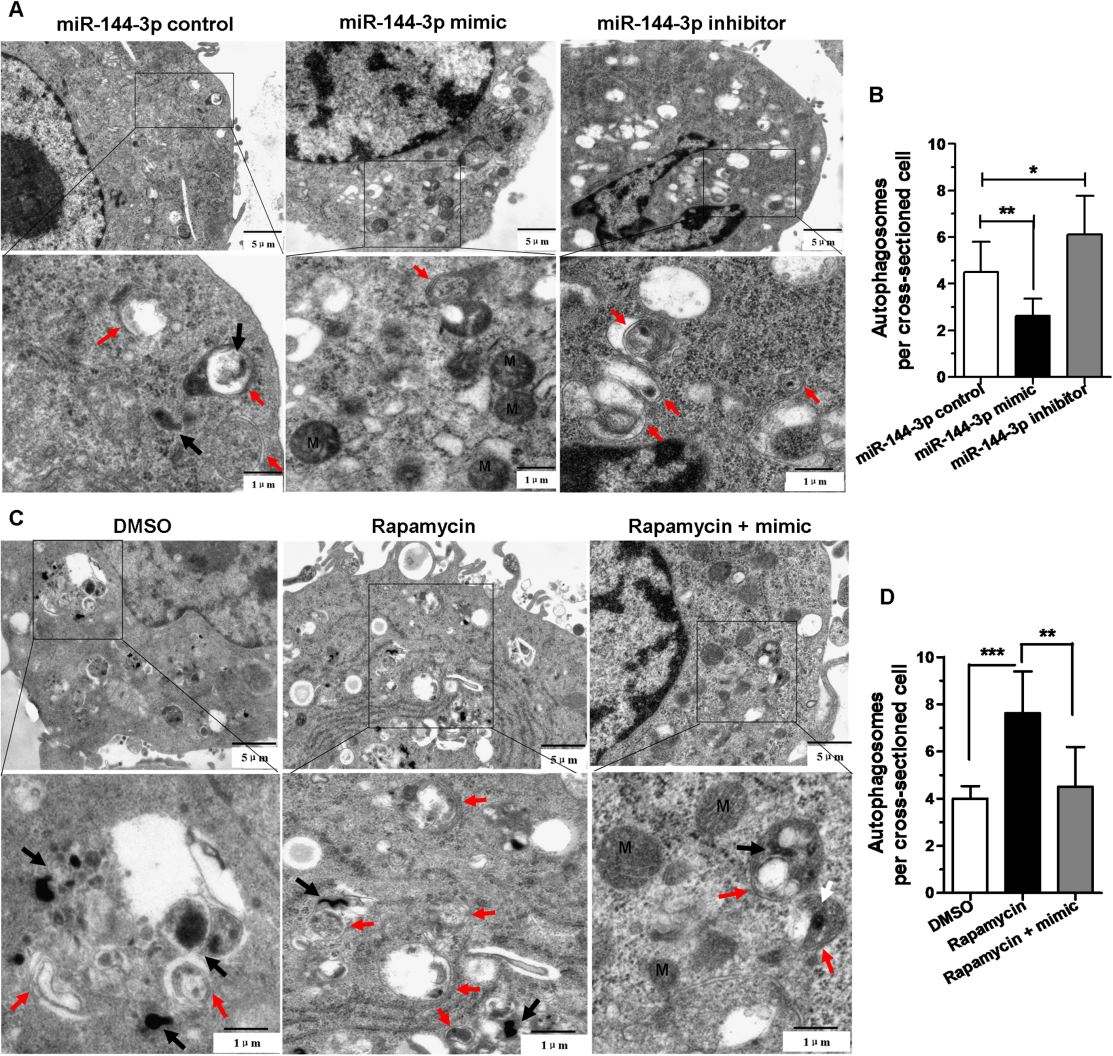
miR-144-3p inhibited autophagosome formation. **(A and B)** Transmission electron microscopy confirms repression of autophagy by miR-144-3p overexpression or knockdown. RAW264.7 cells were transfected with miR-144-3p control, miR-144-3p mimic or miR-144-3p inhibitor for 24 h, and then infected with BCG for 24 h. M, mitochondrion. Autophagosomes (denoted by red arrows). The number of autophagosomes per cross-sectioned cell was counted (15 cells per group counted by TEM). *p<0.05, **p<0.01. **(C and D)** Overexpression of miR-144-3p could attenuate induction of autophagy by rapamycin. The BCG-challenged RAW264.7 cells were transfected with miR-144-3p mimics, and then treated with 50 μg/ml rapamycin for 2 h. Meanwhile the BCG-challenged RAW264.7 cells were treated with rapamycin or 0.1% DMSO as controls. BCG bacteria (denoted by black arrows). M, mitochondrion. Autophagosomes (denoted by red arrows). The number of autophagosomes per cross-sectioned cell was counted (15 cells per group counted by TEM). **p<0.01, ***p<0.001.

For better determination of the inhibitory effect of miR-144-3p on autophagy, TEM was utilized to detect autophagosomes. We found that there were less autophagosomes in RAW264.7 cells transfected with miR-144-3p mimics than cells transfected with miR-144-3p control. In addition, the RAW264.7 cells transfected with miR-144-3p inhibitors contained more autophagosomes than those cells transfected with miR-144-3p control (Fig 4A and 4B). Furthermore, the rapamycin-treated cells transfected with miR-144-3p mimics displayed less autophagosomes than those cells treated only with rapamycin (Fig 4C and 4D). These results indicated that overexpression of miR-144-3p could inhibit autophagic response in macrophages and down-regulation of miR-144-3p could promote autophagic response. Besides, miR-144-3p could attenuate induction of autophagy by rapamycin.

### MiR-144-3p enhances intracellular BCG survival in macrophages

**Fig 5:**
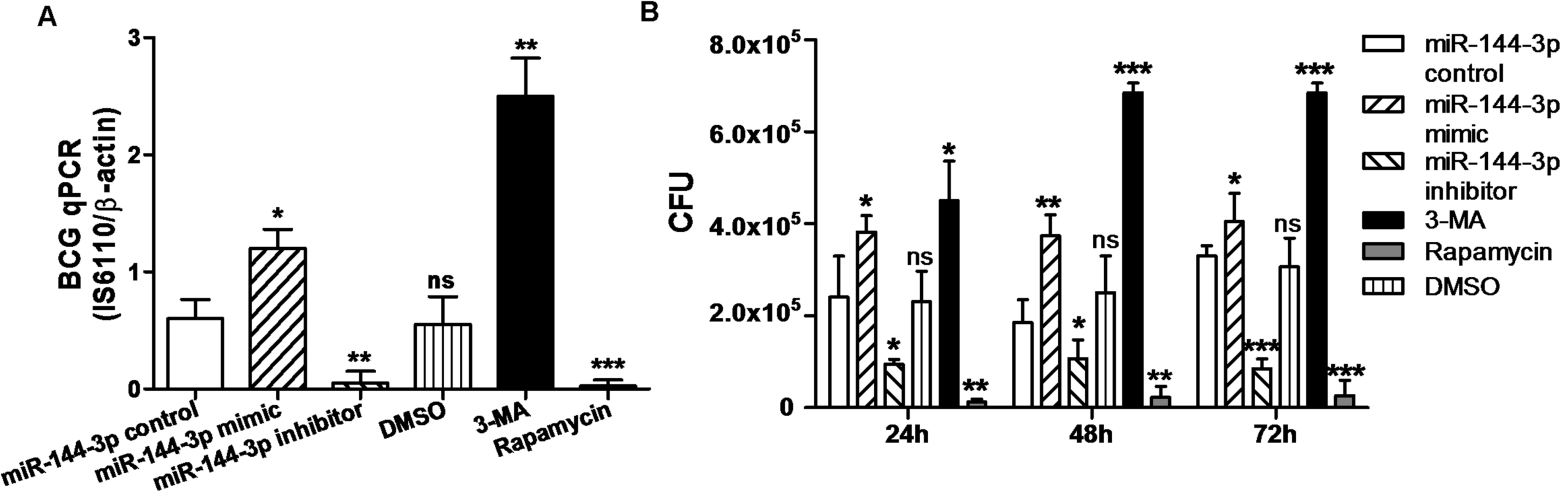
Intracellular BCG survival analyzed by qPCR and CFU assay. **(A)** The RAW264.7 cells were transfected with miR-144-3p control, miR-144-3p mimic or inhibitor, or were treated with rapamycin or 3-MA, and then infected with BCG for 24 h. The DNA of intracellular BCG was determined by using a qPCR assay. Cells trated with rapamycin or 3-MA were used as a positive or negtive control of autophagy. *p<0.05, **p<0.01, ***p<0.001. **(B)** The RAW264.7 cells were transfected with miR-144-3p control, miR-144-3p mimic or miR-144-3p inhibitor, or were treated with rapamycin or 3-MA. After that, the RAW264.7 cells were infected with BCG for 24 h, 48 h and 72 h. Intracellular BCG survival was evaluated by CFU assay. *p<0.05, **p<0.01, ***p<0.001.

In order to further determine whether miR-144-3p can enhance BCG survival in macrophages, the abundance of intracellular bacilli was determined by qPCR assay and Colony-Forming Unit (CFU) assay. With this in mind, the RAW264.7 cells were transiently transfected with miR-144-3p control, miR-144-3p mimic or inhibitor for 24 h before they were infected with BCG. Meanwhile the RAW264.7 cells were treated with rapamycin or 3-MA as controls, respectively. The qPCR assay showed that the abundance of the IS6110 DNA sequence in RAW264.7 cells transfected with miR-144-3p mimic or 3-MA was greater than that in RAW264.7 cells transfected with miR-144-3p control. Additionally, transfection with miR-144-3p inhibitor reduced the abundance of the IS6110 DNA sequence in RAW264.7 cells compared with transfection with miR-144-3p control (Fig 5A). Beside, the BCG viability in macrophages was also tested by CFU assay. Transfection of RAW264.7 cells with miR-144-3p mimic promoted intracellular BCG growth. However, transfection with miR-144-3p inhibitor inhibited BCG growth in macrophages (Fig 5B). Together, these results showed that miR-144-3p enhanced BCG survival in macrophages by inhibiting autophagy.

## Discussion

**Fig 6:**
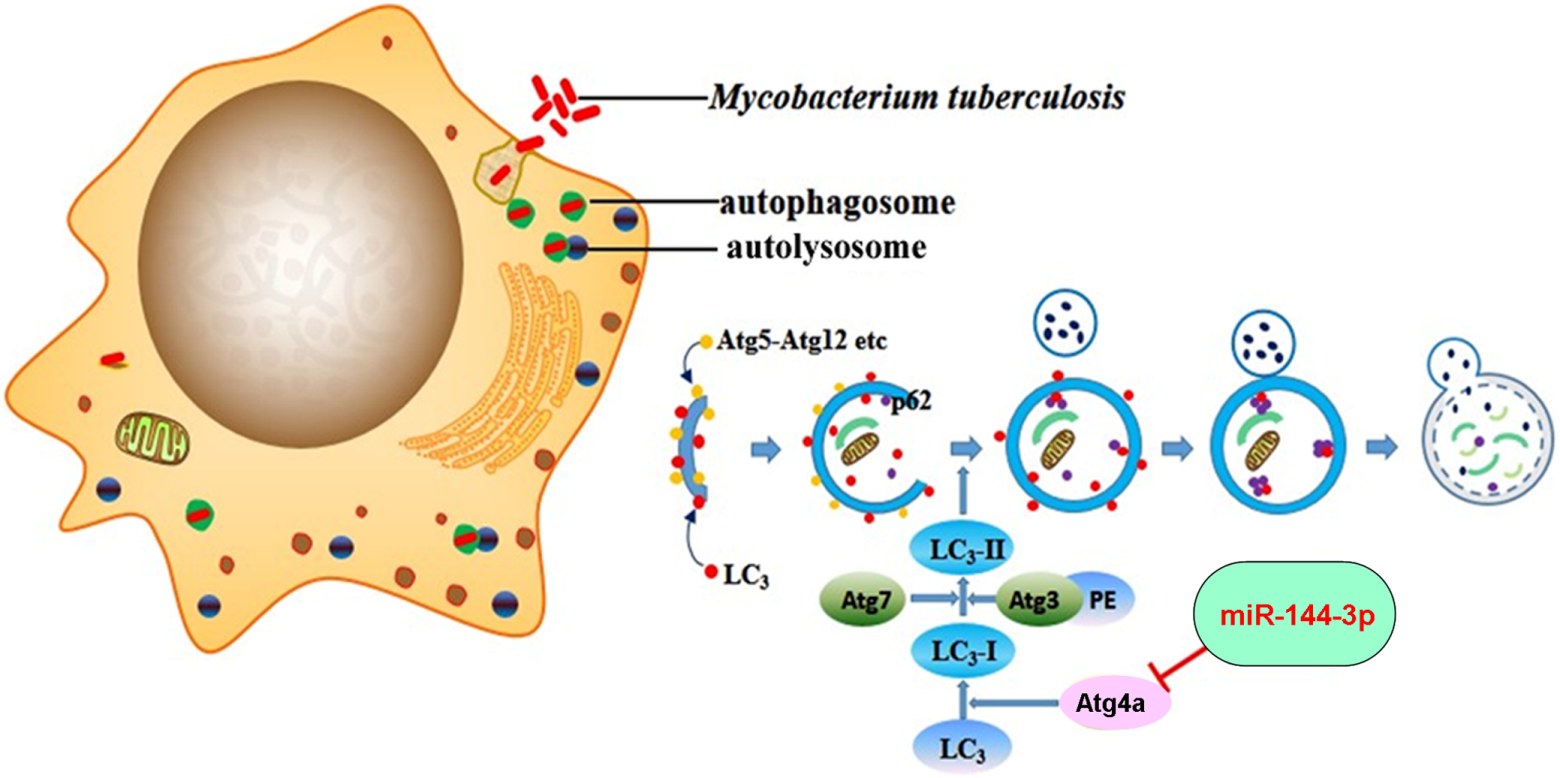
Schematic representation of miR-144-3p inhibiting autophagy activation during mycobacterial infection by targeting ATG4a. MiR-144-3p overexpression in macrophages directly represses ATG4a by binding to its 3’UTR, which in turn inhibits the formation of autophagosomes in macrophages and increases the survival rate of intracellular mycobacteria.

Autophagy has been demonstrated to play an essential role in activating the antimicrobial host defense against mycobacterial infection. However, the molecular mechanism involved in the autophagy-mediated control of mycobacteria remains largely unclear and is a focus of current studies. Increasing numbers of miRNAs have been shown as regulators of autophagy pathways [38]. Recent studies have shown that several miRNAs, such as miR-20a [30], miR-155 [23] and miR-125a-3p [32], can regulate host autophagic response against mycobacteria. However, the precise mechanism of miRNA-regulated autophagy during *M*. *tuberculosis* infection is far from clear. Here we demonstrate that miR-144-3p can regulate the expression of ATG4a, thereby modulating activation of the autophagic pathway and affecting antimycobacterial immunity. We found that miR-144-3p modulated the induction of autophagy and the maturation of autophagosomes during BCG infection by inhibiting ATG4a protein expression (Fig 6) and this effect is mediated through the miR-144-3p complementary sequences contained in the 3’ UTR of ATG4a. In our study, BCG infection could up-regulate miR-144-3p (Fig 1), and miR-144-3p could down-regulate ATG4a (Fig 2D and 2E). However, BCG infection significantly increased ATG4a at protein level (Fig 3E). We speculated that the up-regulated miR-144-3p could inhibit the expression of ATG4a in BCG-infected macrophages, this adjusting process may lag behind autophagy induced by BCG. Moreover, the part of downregulated ATG4a by miR-144-3p may be not sufficient to counteract the upregulated ATG4a induced by BCG. Therefore, despite overexpression of miR-144-3p in BCG-infected macrophages, autophagy still increased. As one of the factors, overexpression of miR-144-3p may slightly continue to suppress ATG4a expression for a long time, leading to inhibiting autophagic responses and increasing intracellular survival of BCG.

We examined BCG-challenged macrophage-like RAW264.7 cells, and found that miR-144-3p expression was gradually increased by the stimulation of BCG in a time- and dose-dependent manner (Fig 1A and 1B). In addition, recent studies have revealed that the expression levels of sputum and serum miR-144 in the tuberculosis patients were both higher than those of the healthy controls [26]. Many studies have confirmed that miR-144-3p plays important roles in many different biological pathways involving inflammatory responses [39], oncogenicity [40] and cell proliferation [41], and mitochondrial function [42]. Autophagy is widely considered as a crucial element of the innate immune response against various invading pathogens, including mycobacteria [43]. However, the exact role of miR-144-3p in elimination of intracellular bacterial pathogens mediated by autophagy needs to be further revealed. Our data provide evidence for an important role for miR-144-3p in regulating ATG4a expression (Fig 2). We found that the overexpression of miR-144-3p suppressed ATG4a protein expression, whereas transfection of cells with mir-144-3p inhibitors resulted in an increase in ATG4a expression in BCG–infected macrophages. Two ubiquitination-like pathways play very important roles in the formation of autophagic vesicle and membrane elongation [44]. The covalent conjugation of the yeast autophagy protein ATG8 [45], or its mammalian homologs MAP1LC3 (or simply LC3) is the end point of these reactions. ATG4 enzymes can cut off the C-terminal last five amino acids of LC3 and expose a critical glycine residue of LC3 [46, 47]. The cyclic utilization of LC3 is critical for the sustained operation of antophagy. Furthermore, ATG4a enzymes play key roles in the deconjugation of LC3 proteins from lipids on the autophagic vesicle surface [48]. It had been reported that autophagy could be bloked by knockdown of ATG4 gene in many different experimental systems [49]. Our data suggest that miR-144-3p is involved in targeting ATG4a to modulate its unique function in autophagy induction during BCG infection.

Whether miR-144-3p influences the activation of autophagy was further analyzed by various experimental methods. In the present study, we demonstrated that miR-144-3p inhibited the processing of LC3 and the accumulation of LC3-II puncta in both BCG-challenged and unchallenged RAW264.7 cells (Fig 3A, 3B and 3C), indicating that miR-144-3p inhibited the autophagic response in macrophages and contributed to intracellular bacterial survival. Moreover, transmission electron microscopy confirms repression of autophagy by miR-144-3p overexpression. LysoTracker Red is a deep red-fluorescent dye for labeling and tracking acid compartments in live cells. The acid compartments tracked by Lysotracker RED contain lysosomes and autolysosomes. Therefore, the number of red spots produced by Lysotracker RED can reflect the intensity of autophagy to some extent. Compared with BCG-infected RAW264.7 cells transfected with miR-144-3p control, there were less red spots in BCG-infected RAW264.7 cells transfection with miR-144-3p mimics or CQ, indicating that overexpression of miR-144-3p could inhibit the formation of lysosome or autolysosome just as CQ could (Fig 3F). Beside, the miR-144-3p inhibitors just like rapamycin which was an autophagy inducer could promote the formation of lysosomes and autolysosomes. Moreover, detection of autophagosomes in cellular cross-section revealed a significant reduction upon miR-144-3p overexpression, relative to the control (Fig 4A, 4B, 4C and 4D). In addition, we found that miR-144-3p significantly promoted the survival of intracellular BCG in RAW264.7 cells. However, miR-144-3p inhibitor reduced bacterial survival of BCG in RAW264.7 cells (Fig 5A and 5B). Therefore, upregulation of miR-144-3p following BCG infection might limit the amplitude of autophagic and autolysosome maturation so that BCG could survive in macrophages. During *M*. *tuberculosis* infection, *M*. *tuberculosis* is able to arrest processes of phagosomal maturation and acidification in macrophages [50]. Many of current studies focus on promoting the elimination of intracellular mycobacteria by clarifying the molecular mechanisms of the antibacterial autophagy that overcomes phagosome maturation block induced by *M*. *tuberculosis* [51]. Our finding that BCG infection caused miR-144-3p induction which in turn suppressed phagosomal maturation and antimycobacterial responses in macrophages by targeting ATG4a suggests that inhibition of autophagy by miR-144-3p is very likely involved in promoting mycobacterial survival.

Taken together, our data reveal a novel role of miR-144-3p in modulating host immunity after BCG infection by controlling ATG4a production. Besides, the findings open a new avenue of research on the molecular mechanism of miRNAs to regulating autophagy by targeting key autophagy-related genes such as ATG4a. Understanding the details of specific miRNAs regulation of autophagy may provide insight into the pathogenesis of various infectious diseases through regulating autophagy process. Furthermore, these studies may shed light on anti-tuberculosis therapeutics, because a range of targeted drugs used to increase antimycobacterial effect by activating autophagy are closely related with host defenses against *M*. *tuberculosis*.

## Supporting information

S1 Table Primers used in this study. (DOC)

S1 Fig miRNA expression profile in dendritic cell (DC) infected with BCG. A heat-map (partial results) of the up-regulated and down-regulated miRNAs in DC by microarray analysis. MiRNAs that Foreground-Background intensities are smaller than 50 in all samples have been excluded. MiR-144-3p expression is up-regulated in DC infected with BCG (DC3) compared with normal control DC (DC1).
(TIF)

S1 Text Supplemental materials and methods. (DOC)
