# Supplementary material for: MicroRNA-144-3p inhibits autophagy activation and enhances Bacillus Calmette-Guérin infection by targeting ATG4a in RAW264.7 macrophage cells
Source: PLoS One. 2017 Jun 21;12(6):e0179772. doi: 10.1371/journal.pone.0179772 (PMC5479589; doi:10.1371/journal.pone.0179772)
Supplement: S1 Table — (DOC) [file pone.0179772.s001.doc]

**S1 Table. Primers used in this study.**

| No | Genes | Forward primer (5’-3’) | Reverse primer (5’-3’) |
| --- | --- | --- | --- |
| 1 | miR-144-3p | 5’-ACACTCCAGCTGGGTACAGTATAGATGATGTACT-3’ | 5’-CTCAACTGGTGTCGTGGA-5’ |
| 2 | IS6110 | 5’-GGACGGAAACTTGAACACG-3’ | 5’-TCTGACGACCTGATGATTGG-3’ |
| 3 | β-actin | 5’-CAAGTCATCACTATTGGCAACGA-3’ | 5’-CCAAGAAGGAAGGCTGGAAAA-3’ |
| 4 | ATG4a 3' UTR(WT) | 5’-CCCAAGCTTGGttagaactcagtgaggtgg-3’ | 5’-CGACGCGTCAAAGTATAATTCAAACAAT-3’ |
| 5 | ATG4a 3' UTR(MT) | 5’-CCCAAGCTTGGttagaactcagtgaggtgg-3’ | 5’-CGACGCGTCACAGTACTGATTCAAACAAT-3’ |
