# Supplementary figures and images for: MicroRNA-144-3p inhibits autophagy activation and enhances Bacillus Calmette-Guérin infection by targeting ATG4a in RAW264.7 macrophage cells
Source: PLoS One. 2017 Jun 21;12(6):e0179772. doi: 10.1371/journal.pone.0179772 (PMC5479589; doi:10.1371/journal.pone.0179772)

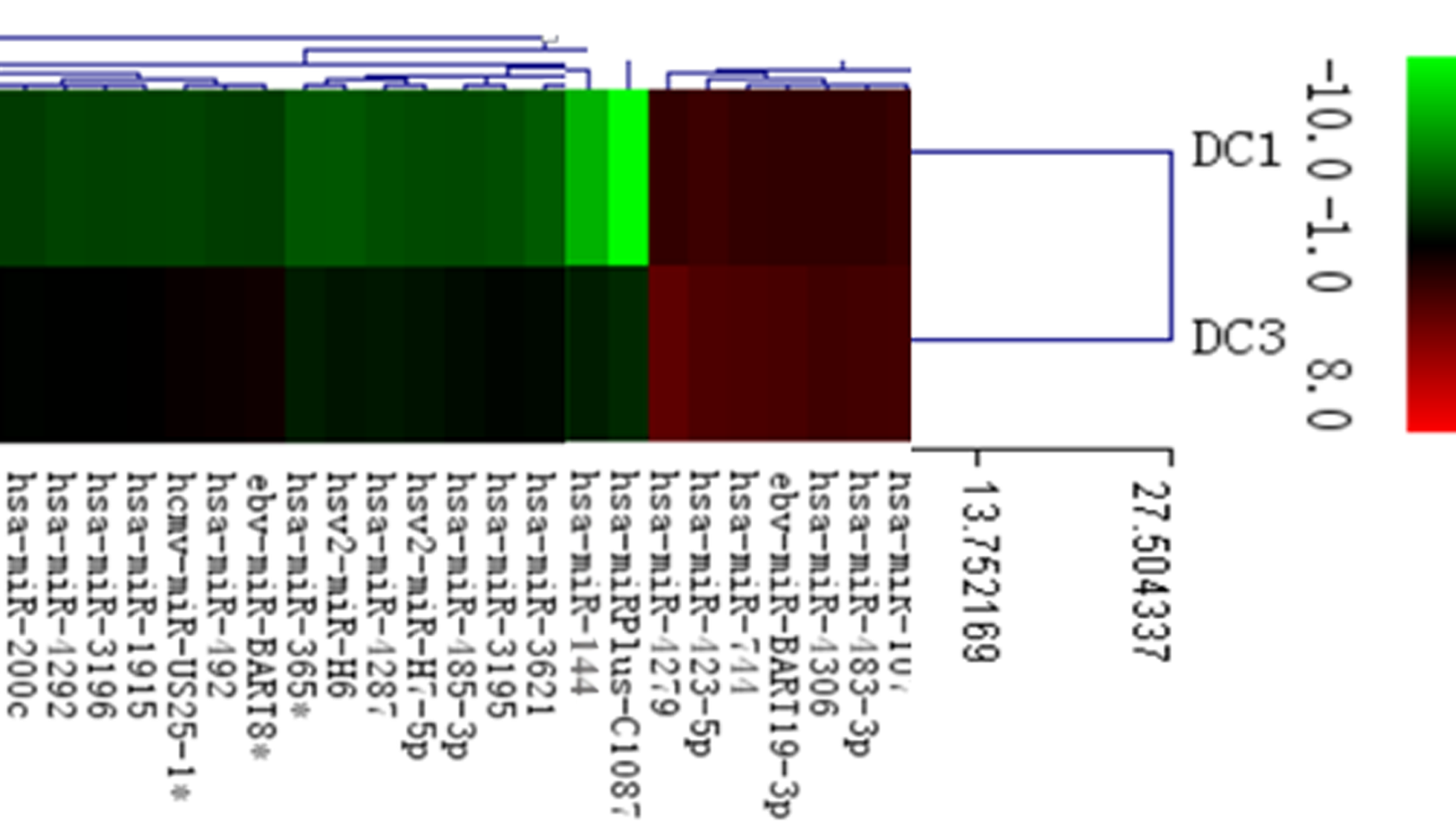

Supplement: S1 Fig — A heat-map (partial results) of the up-regulated and down-regulated miRNAs in DC by microarray analysis. MiRNAs that Foreground-Background intensities are smaller than 50 in all samples have been excluded. MiR-144-3p expression is up-regulated in DC infected with BCG (DC3) compared with normal control DC (DC1). (TIF) [file pone.0179772.s002.tif]
