# Supplementary material for: MicroRNA-144-3p inhibits autophagy activation and enhances Bacillus Calmette-Guérin infection by targeting ATG4a in RAW264.7 macrophage cells
Source: PLoS One. 2017 Jun 21;12(6):e0179772. doi: 10.1371/journal.pone.0179772 (PMC5479589; doi:10.1371/journal.pone.0179772)
Supplement: S1 Text — (DOC) [file pone.0179772.s003.doc]

**Analysis of miRNA expression profiling in** **BCG-infected dendritic cells by**

**miRNA microarray**

The 6th generation of miRCURYTM LNA Array (v.16.0) (Exiqon) contains more than 1891 capture probes, covering all human, mouse and rat microRNAs annotated in miRBase 16.0, as well as all viral microRNAs related to these species. In addition, this array contains capture probes for 66 new miRPlus™ human microRNAs.

**RNA extraction**

Total RNA of BCG-infected dendritic cells or normal dendritic cells was isolated using TRIzol (Invitrogen) and miRNeasy mini kit (QIAGEN) according to manufacturer’s instructions, which efficiently recovered all RNA species, including miRNAs. RNA quality and quantity was measured by using nanodrop spectrophotometer (ND-1000, Nanodrop Technologies) and RNA Integrity was determined by gel electrophoresis. Please refer to “RNA_QC.pdf”.

**RNA labeling**

After RNA isolation from the samples, the miRCURY™ Hy3™/Hy5™ Power labeling kit (Exiqon, Vedbaek, Denmark) was used according to the manufacturer’s guideline for miRNA labelling. One microgram of each sample was 3'-end-labeled with Hy3TM fluorescent label, using T4 RNA ligase by the following procedure: RNA in 2.0 μL of water was combined with 1.0 μL of CIP buffer and CIP (Exiqon). The mixture was incubated for 30 min at 37°C, and was terminated by incubation for 5 min at 95°C. Then 3.0 μL of labeling buffer, 1.5 μL of fluorescent label (Hy3TM), 2.0 μL of DMSO, 2.0 μL of labeling enzyme were added into the mixture. The labeling reaction was incubated for 1 h at 16°C, and terminated by incubation for 15 min at 65°C.

**Array hybridization**

After stopping the labeling procedure, the Hy3TM-labeled samples were hybridized on the miRCURYTM LNA Array (v.16.0) (Exiqon) according to array manual. The total 25 μL mixture from Hy3TM-labeled samples with 25 μL hybridization buffer were first denatured for 2 min at 95°C, incubated on ice for 2 min and then hybridized to the microarray for 16–20 h at 56°C in a 12-Bay Hybridization Systems (Hybridization System - Nimblegen Systems, Inc., Madison, WI, USA), which provides an active mixing action and constant incubation temperature to improve hybridization uniformity and enhance signal. Following hybridization, the slides were achieved, washed several times using Wash buffer kit (Exiqon), and finally dried by centrifugation for 5 min at 400 rpm. Then the slides were scanned using the Axon GenePix 4000B microarray scanner (Axon Instruments, Foster City, CA).

**Data analysis**

Scanned images were then imported into GenePix Pro 6.0 software (Axon) for grid alignment and data extraction. Replicated miRNAs were averaged and miRNAs that intensities>=50 in all samples were chosen for calculating normalization factor. Expressed data were normalized using the Median normalization. After normalization, differentially expressed miRNAs were identified through Fold Change filtering. Hierarchical clustering was performed using MEV software (v4.6, TIGR).
